# The influence of bootleg innovation on individual innovation performance: The mediating effect of cognitive flexibility and the moderating effect of leadership’s emotional intelligence

**DOI:** 10.1371/journal.pone.0296782

**Published:** 2024-02-02

**Authors:** Xiaoxiao Gao, Longmei Wang, Lei Lu, Weilin Wu

**Affiliations:** 1 School of Business, Shenzhen Institute of Technology, Shenzhen, China; 2 Zhongshan Polytechnic, Zhongshan, China; 3 School of Psychological and Cognitive Sciences, Beijing Key Laboratory of Behavior and Mental Health, Peking University, Beijing, China; 4 School of Economics, Institute of China Common Prosperity Research, Jiaxing University, Jiaxing, Zhejiang, China; Czestochowa University of Technology: Politechnika Czestochowska, POLAND

## Abstract

Based Correctly handling the creativity of employees who have not been adopted is not only conducive to continuously stimulating employees’ creativity and improving individual innovation performance, but also conducive to making the best use of organizational resources. This study integrates conservation of resource theory (COR) and social information processing theory to explore the influence of bootleg innovation behavior in organizations on individual innovation performance, as well as the mediating role of cognitive flexibility and the moderating role of leadership emotional intelligence. A three-stage time-lagged research design is used to obtain a valid sample of 327 employees from China. The PROCESS macro for SPSS was applied to test the hypothesized relationships. Findings demonstrated that bootleg innovation is positively related to individual innovation performance; cognitive flexibility mediates the relationship between bootleg innovation and individual innovation performance. Moreover, leadership emotional intelligence moderates the relationship between bootleg innovation and individual innovation performance and between bootleg innovation and cognitive flexibility and between cognitive flexibility and individual innovation performance respectively. The conclusion of the study not only provides a theoretical basis for individuals and leaders to deal with employees’ creative abortion, but also provides a new thinking mode for how to maximize the effectiveness of unaccepted ideas and promote individual innovation performance.

## 1 Introduction

In organizations, innovation plays a crucial role in sustaining organizational efficiency, fostering growth, and enhancing performance [1, 2]. Consequently, enhancing employees’ innovative capabilities and fostering their innovative mindset has been a central concern for organizations and a subject of extensive scholarly inquiry [3–6]. While organizations encourage employees to proactively propose innovative solutions, the implementation of such solutions is limited due to resource constraints [7]. To ensure that resources are allocated to mature and strategically aligned innovation plans, organizations establish rigorous audit programs, and may not readily accept individual employees’ high-risk innovation behaviors [8]. Despite facing rejection, some employees persist in pursuing their innovative ideas covertly, firmly believing in their feasibility and potential benefits for the organization. Even if the innovative proposal is rejected, individuals may still pursue it covertly, a behavior known as bootleg innovation [9]. This phenomenon is observed periodically within organizations, with approximately 5–10% of individuals in research and development teams engaging in bootleg innovation [10]. For instance, in 2006, Wang Xiaochuan, the vice president of Sohu, identified untapped business opportunities in the browser market and clandestinely collaborated with colleagues to develop and research a browser without authorization. After three years of effort, the Sogou browser was successfully launched [11]. The prevalence of bootleg innovation is attributed to organizations prioritizing innovation outcomes over the innovation process, serving as a motivating factor for employees to persist with their innovative ideas [12]. Consequently, the study of bootleg innovation has gained prominence, with scholars conducting empirical research to examine its impact [13–15]. Existing literature presents varying perspectives on the relationship between bootleg innovation and individual performance, with some scholars demonstrating positive, negative, and inverted U-shaped relationships [12, 16]. However, the impact of bootleg innovation on individual innovation performance is influenced by multiple factors, including individual, leadership, and organizational factors [17]. While previous studies have explored the impact of bootleg innovation from different angles, the understanding of its impact path remains limited, failing to fully elucidate the connection between deviant innovation and individual innovation performance.

Bootleg innovation represents an unconventional thinking approach, and not all individuals will enhance their performance through this method [18]. Research suggests that employees’ high work autonomy and creativity are pivotal drivers of bootleg innovation [19]. Individual factors play a crucial role in shaping deviant innovation, particularly in the context of organizational pressures for innovation performance and limited resources. Under such circumstances, individuals must exert considerable effort to address challenges and develop coping mechanisms, with cognitive flexibility being one such mechanism [20]. Cognitive flexibility, characterized by the ability to adapt to complex situations through cognitive processing strategies, enables employees to confidently navigate new situations [21, 22]. An individual’s open-mindedness and flexible thinking facilitate their acceptance and rational response to diverse situations, fostering quick-wittedness, self-confidence, and insight [23, 24]. This flexible cognitive approach contributes to the generation of novel ideas [24]. According to social information processing theory, individuals continuously adjust their attitudes, behaviors, and beliefs based on their social environment and past experiences, and a flexible thinking mode facilitates the management of work-related challenges, ultimately leading to improved performance [25].

Organizations must carefully balance the level of autonomy granted to employees in conducting research, as excessive freedom may lead to a disconnect between research activities and organizational goals, while overly restrictive oversight may stifle employees’ research and development capabilities [12]. Effectively harnessing employees’ creativity within limited resources and authority is a critical challenge, with leadership exerting a significant influence on employees’ creative deviance and performance [26]. Studies have highlighted the need for employees to adapt their behaviors in response to environmental changes and innovation processes, with leaders playing a pivotal role in this dynamic by understanding and managing emotions, which in turn impacts team performance through interpersonal interactions [27–29]. Negative emotions can lead to conflicts and adverse outcomes [30], while leaders with high emotional intelligence have been shown to understand and respond to employees’ emotions effectively, motivating team members and fostering overall team output [31, 32]. However, empirical research on the relationship between leadership emotional intelligence and bootleg innovation, and its impact on individual innovation performance, remains limited. Therefore, further investigation is needed to clarify the characteristics and boundary conditions of bootleg innovation.

To address these gaps, this study aims to examine the influence of bootleg innovation on individual innovation performance, drawing on resource conservation theory and social information processing theory. The study will introduce individual factors such as cognitive flexibility and leadership factors like emotional intelligence as moderator variables, exploring their effects on bootleg innovation and individual innovation performance, as well as their boundary conditions. Ultimately, this research seeks to enhance understanding of the relationship between bootleg innovation and innovation performance, offering practical insights for corporate managers to effectively leverage the positive effects of bootleg innovation and mitigate its negative impact.

**Fig 1:**
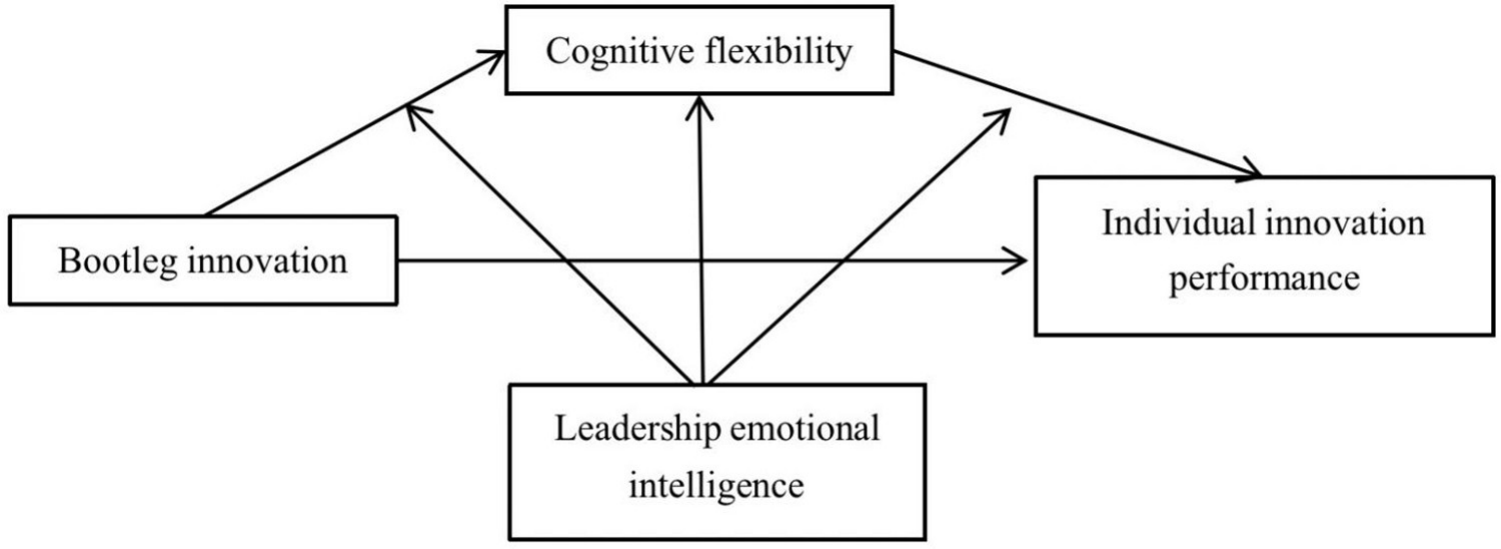
Model assumption.

To sum up, the model hypothesis proposed in this study is shown in Fig 1.

## 2 Theoretical basis and research hypotheses

### 2.1 Bootleg innovation and individual innovation performance

Fierce competition and a constantly changing business environment compel organizations to ensure their survival and growth through ongoing innovation [33]. Organizations encourage active employee participation in innovation [34]. However, due to limited resources, only a small number of ideas can be supported to avoid creating resource constraints, which may lead to deviant behavior in innovation activities [7]. Previous literature lacks a precise definition of bootleg innovation. Augsdorfer [9] defines bootleg innovation as the spontaneous innovative behavior of grassroots employees, formed without informing management, and expected to benefit the organization, characterized by autonomy and concealment. Mainemelis [7] views deviant innovation as behavior that defies organizational management and authority, persistently explores previous ideas, and highlights violations of leadership directives. Warren [35] holds a different perspective, considering behaviors that violate organizational norms and enhance the organization’s well-being as bootleg innovation. This paper adopts Augsdorfer’s viewpoint, defining bootleg innovation as individual employee behavior aimed at benefiting the organization without informing managers, based on previous research.

Existing studies have demonstrated that rigid work structures can stifle the emergence of innovative ideas within organizations [36]. In enterprises where innovation is considered a vital resource for survival, the clash of new ideas can stimulate employees’ innovative abilities. New ideas stem from individual bottom-up initiatives, rather than structured decision-making or management strategies [37]. When individuals break free from organizational norms and gain flexibility, they can explore uncharted territories and gain an exploratory advantage [38, 39]. Harnessing strengths can enable employees to exhibit more creative thinking at work, a crucial factor in enhancing creativity and innovation performance [16]. Individuals engage in deviant innovation for two reasons: (1) the organization delays the evaluation of new ideas through bootleg innovation, providing individuals with ample time to refine their ideas, enhance the feasibility of creative plans, and reduce the likelihood of premature exposure and rejection of their plans [40, 41]. (2) The organization may reject an idea, but the individual still perceives it as having research value. Bootleg innovation can positively impact team performance through a "maximum output with minimum input" approach [42]. Regardless of the reason, the delayed impact of bootleg innovation can mitigate the risk of an idea being rejected by the organization. In fact, some level of risk and uncertainty in research and development is often a prerequisite for genuinely novel ideas [12]. The diverse suggestions or ideas conveyed through bootleg innovation behaviors help compensate for cognitive and informational blind spots [16]. Differing from mechanical execution, deviant innovation represents new thinking and methods. This exploratory process expands individual cognitive horizons and enables the acquisition of rich information, which is pivotal for enhancing innovation performance.

According to the COR theory, individuals are inclined to strive for the acquisition, maintenance, cultivation, and protection of resources they value [43]. Consequently, prematurely presenting ideas may not only attract attention but also heighten the risk of rejection by the organization. Subsequent rejection can lead to frustration, hindering the individual’s future work [44]. By delaying the proposal, individuals can engage in thorough exploration in a less restrictive environment until the opportune moment, thereby enhancing the likelihood of organizational acceptance and bolstering innovation performance [45]. Simultaneously, even if research reveals inherent limitations in creativity that prevent advancement, it will not overly burden employees with attention and frustration, which could negatively impact future work [16].

Therefore, the following hypotheses are proposed:

*H1*: *Bootleg innovation is positively related to individual innovation performance*.

### 2.2 Mediating role of cognitive flexibility

Cognitive flexibility is the capacity of an individual to adjust cognitive processing strategies in order to effectively navigate unfamiliar situations [46]. Individuals with high cognitive flexibility not only demonstrate adaptability in response to changing circumstances, but also exhibit a belief in their ability to further enhance their adaptability [47]. This ability is considered a significant manifestation of individual intelligence and reflects an individual’s capacity to acclimate to their surroundings [48]. Employees with elevated cognitive flexibility possess strong insight and self-assurance, enabling them to swiftly pivot in response to environmental changes and identify new resources to address challenges [24]. Conversely, individuals with low cognitive flexibility tend to adhere to rigid thought patterns, employing conservative processing methods to minimize risk and avoid failure [49]. Bootleg innovation, a process that challenges employees’ thinking and knowledge reserves, necessitates continual shifts in thinking patterns and the practical application of acquired knowledge [50]. Through repeated simulations, experiments, and tests, individuals are encouraged to modify their cognition, thereby deepening and reinforcing their knowledge through a cycle of theory, practice, and theory, ultimately enhancing their cognitive flexibility [51]. Furthermore, research has indicated that cognitive flexibility influences an individual’s input in innovation, subsequently impacting innovation performance. A high level of cognitive flexibility can facilitate a flexible cognitive process, enabling individuals to swiftly overcome challenges by continuously altering their thought processes [47]. Cognitive flexibility encourages heightened awareness and a propensity for creativity and innovation [50]. When individuals with elevated cognitive flexibility encounter obstacles in the process of deviant innovation, they leverage their acute sense of opportunities to actively adjust their thinking and strategies, leading to the attainment of superior individual innovation performance [50].

According to the COR theory, individuals are motivated to acquire new resources in order to cope with future changes while ensuring the preservation of existing resources in the face of stressful situations such as bootleg innovation [43]. Research has indicated that individuals with high cognitive flexibility often exhibit divergent thinking and are capable of generating multiple solutions from various perspectives to address problems, thereby enhancing creativity in scientific research [52]. Furthermore, these individuals actively pursue resources to implement innovative ideas [53] and demonstrate a strong confidence in their ability to solve scientific research problems [46], which serves as a crucial factor in achieving success in innovation and individual performance in innovation.

Therefore, the following hypotheses are proposed:

*H2*: *Cognitive flexibility mediates the relationship between bootleg innovation and individual innovation performance*.

### 2.3 Moderating role of leadership emotional intelligence

According to the concept of "delayed disclosure advantage," employees have the opportunity to gather sufficient evidence before formally presenting an innovation plan through deviant innovation. This approach can enhance the likelihood of acceptance and improve individual performance [7]. Previous research has demonstrated that deviant innovation can effectively circumvent excessive organizational intervention, challenge conventional thinking, and yield unexpected creative outcomes [31]. However, bootleg innovation is a challenging and arduous process, and relying solely on individual frustration self-regulation may seem ineffective. Support and encouragement from the external environment can provide employees with additional motivation [54]. Studies have indicated that within organizations, leaders with high emotional intelligence excel at motivating subordinates [55], and have a positive influence on subordinates’ task performance and organizational citizenship behavior [56, 57].

Emotional intelligence encompasses the capacity of individuals to effectively manage their own emotions and accurately discern and respond to the emotions of others within a given context [57]. Leaders with high emotional intelligence possess the ability to regulate both their own emotions and those of others, fostering a supportive and collaborative atmosphere within the organization [28]. This enables individuals facing challenges to discreetly seek assistance from colleagues or leaders, facilitating expedited problem-solving. Additionally, leaders with high emotional intelligence are adept at promptly recognizing shifts in employees’ emotions. When employees exhibit signs of distress due to difficulties in their innovative work, leaders can provide empathetic and constructive feedback, such as appropriate incentives [31], and effectively modulate employees’ emotions and behaviors [28]. This approach aids in resolving employees’ challenges in a more positive state, thereby expediting the achievement of innovation objectives.

The social information processing theory posits that individuals’ behaviors and attitudes are influenced by their environment, and they continuously adapt their beliefs and behaviors by processing environmental information [25]. Within an organizational context, leaders and colleagues serve as primary sources of information for employees. Prior research has suggested that leaders’ emotional intelligence can have a cascading impact on their subordinates’ emotions, leading subordinates to model their work behaviors based on observed changes in their leader’s emotions [58]. Increasingly, studies on emotional intelligence have demonstrated that leaders’ emotional intelligence significantly influences both their own and their subordinates’ work outcomes [59]. Leaders can transmit their emotions to employees through language and behavior, thereby influencing employees’ emotional states [60]. Leaders with higher emotional intelligence are more adept at managing their own emotions, such as anger and negativity, compared to those with lower emotional intelligence, thus preventing emotional spillover to subordinates [6]. In instances where employees experience negative emotions, leaders can encourage and empower employees, bolster their self-confidence, and foster the generation of innovative performance. Therefore, we hypothesize the following:

*H3*: *Leadership emotional intelligence moderates the relationship between bootleg innovation and individual innovation performance such that the effect is stronger for more leadership emotional intelligence than less leadership emotional intelligence*.

Bootleg innovations aim to increase innovation performance and conduct research privately without organizational support [12]. Bootleg innovations have a higher chance of designing revolutionary products [7]. Even if it is not successful, it can not only provide experience for follow-up research, but also consume lower organizational costs [61, 62]. This favorable outcome leads employees to continue investing time, energy, and personal resources in innovative research [6]. In the face of complex and changeable problems, maintain a continuous learning state and ensure flexible adaptation to various situations [63]. At the same time, studies have shown that in the workplace, the relationship between leaders and employees will also affect employees’ deviant innovation [64]. Emotional intelligence represents the social skills of leaders and plays a role in the interaction between leaders and employees [65, 66]. Only when a leader’s emotional intelligence maintains a dynamic resonance with the emotional perception of his subordinates can he effectively improve organizational effectiveness [6]. Because of this, employees’ perceptions of the quality of their relationship with leaders create a work environment for employees, which in turn affects the development of certain capabilities, such as cognitive flexibility [67]. According to the theory of social information processing, the environment around an individual will affect the behavior and activities of the individual [25]. When leaders demonstrate higher emotional intelligence, they will handle their relationships with employees with a more accurate ability to perceive, evaluate, and express emotions [68]. By correctly understanding employees’ emotions and giving them appropriate feedback [65], the safe working environment constructed by this harmonious relationship can stimulate employees to generate more inspiration, and they can find new ways to solve problems even in the face of difficulties. Studies have shown that leaders with high emotional intelligence can accurately grasp employees’ emotional states and behavioral responses, and can effectively regulate employees’ emotions [28]. As a result, employees can challenge risks with an optimistic, confident, and flexible attitude and stimulate their creativity [45]. Therefore, this study proposes the following assumptions:

*H4*: *Leadership emotional intelligence moderates the relationship between bootleg innovation and cognitive flexibility such that the effect is stronger for more leadership emotional intelligence than for less leadership emotional intelligence*.

Cognitive flexibility refers to an individual’s ability to think and approach various perspectives in a flexible manner [69]. Research indicates that individuals who engage in brainstorming activities demonstrate higher levels of cognitive flexibility [70]. Furthermore, it has been demonstrated that adopting a broader perspective can facilitate creativity and subsequently enhance innovation performance [71]. In addition to individual traits, leadership qualities also play a significant role in influencing innovation performance [72].

Prior studies have indicated that the emotional intelligence of leaders has a significant impact on both employee behavior and performance [55]. When leaders exhibit higher emotional intelligence, they are better equipped to manage their relationships with employees by accurately perceiving, evaluating, and expressing emotions [68]. Kafetsios et al. [73] discovered a positive correlation between leaders’ use of emotion and job satisfaction and positive emotions among team members. Leaders with high emotional intelligence, based on self-emotional control, are capable of fostering positive emotions [66]. Furthermore, they can effectively regulate employee emotions by accurately understanding their emotions and providing appropriate feedback [28]. A positive work environment, combined with effective communication, can greatly assist employees in coping with challenging situations. In return, employees are motivated to work harder to reciprocate the trust, understanding, and support of their leaders, thus creating a positive reinforcement cycle [55]. Consequently, employees can adopt an optimistic, confident, flexible, and positive attitude towards facing risks, overcoming difficulties, stimulating their creativity, and ultimately enhancing their individual innovation performance [45].

Therefore, this study proposes the following assumptions:

*H5*: *Leadership emotional intelligence moderates the relationship between cognitive flexibility and individual innovation performance such that the effect is stronger for more leadership emotional intelligence than for less leadership emotional intelligence*.

Innovation serves as the primary driver of productivity. The emergence of unconventional innovation is aimed at achieving higher levels of innovative performance [10]. Both employees and leaders aspire to harness the positive aspects of unconventional innovation [16]. For employees, engaging in the process of unconventional innovation and addressing various challenges in a flexible cognitive manner can enhance individual innovative performance [50]. Research has demonstrated that when leaders and employees share compatible cognitive perspectives, employees exhibit improved work behavior and outcomes [74]. High emotional intelligence is a crucial attribute for effective leadership [75]. Leaders with high emotional intelligence can not only provide essential social support to employees but also alleviate their work-related stress [76]. According to social information processing theory, employees’ activities and behaviors in the workplace continually adapt their attitudes under the influence of the organizational environment. In the process of unconventional innovation, employees encounter numerous setbacks. In comparison to leaders with low emotional intelligence, those with high emotional intelligence are more inclined to support others with positive emotional states, offer encouragement through sympathetic and supportive emotional states, and motivate employees to become more resilient in the face of challenges, ultimately leading to higher individual innovative performance [77]. Consequently, this study posits the following assumptions:

Therefore, this study proposes the following assumptions:

*H6*: *Leadership emotional intelligence moderates the indirect effect of bootleg innovation and individual innovation performance via cognitive flexibility; such that the indirect effect will be stronger for more leadership emotional intelligence than for less leadership emotional intelligence*.

## 3 Research methods

### 3.1 Study samples and procedures

In this study, questionnaire survey was used to collect data. According to previous scholars’ opinions and research experience, bootleg innovations mostly occur in companies with high-tech industries [12, 19]. The samples are mainly from 10 high-tech industries in Shanxi province, Guangdong province, Shanghai, Beijing and Macau in China. The main person under investigation is full-time employees and direct supervisors of software development and biopharmaceutical industries. Because this study takes China as the research object, the snowball sampling method is adopted to investigate, and the research shows that this is the most suitable investigation method for China’s research background [78].

This questionnaire is divided into two types, one is the employee questionnaire, and the other is the supervisor questionnaire filled out by the employee’s corresponding direct supervisor. First of all, we selected 10 companies in China’s Shanxi Province, Guangdong Province, Shanghai, Beijing and Macau that fit the research background. We contacted the human resources directors of each company and elaborated on the purpose and methods of our research, and received their support. The Director of Human Resources provides a list of potential investigators and encourages employees and their immediate supervisors to participate in the investigation. Based on the personnel list provided by the Human Resources Director. We randomly selected people based on a ratio of 1:10 between supervisors and employees, distributed questionnaires, and finally returned the questionnaires to us after completing their responses. In order to avoid common method deviation (CMB), this study collected data in three stages [79], taking the last four digits of the respondents’ mobile phone numbers as the matching basis. In order to avoid worries for employees, we emphasize that the questionnaire is anonymous and the results are for research purposes only. So they could be filled in with confidence. The first wave mainly measures bootleg innovation, and demographic variables, which is filled in by employees. Issued on May 15th, 2022, 400 questionnaires were collected from 10 industries. 388 questionnaires were collected on May 20th. Based on the list of HR directors, we categorized the returned questionnaires into company and department categories and coded them. In the second wave, leadership emotional intelligence and cognitive flexibility were measured, which were filled in by employees. Distributed on May 30, 2022, and 365 questionnaires were collected on June 5. Subsequently, we classified the collected questionnaires according to companies and departments. In the third wave, the employee’s individual innovation performance is measured, which is filled in by the employee’s immediate supervisor. We provide the supervisor with a code so that we can know the order of their subordinate list in the questionnaire and facilitate matching. It was distributed on June 15th, 2022, and 338 questionnaires were collected on June 20th. After three stages of data matching, invalid questionnaires were eliminated, and the final number of valid questionnaires was 327, with an effective recovery rate of 82%.

Among them, 74 are female, accounting for 23.63%, and 253 are male, accounting for 76.37%. It can be seen that the number of male in this survey is obviously more than that of female. From the age point of view, the number of people aged 30 and below is 169, accounting for 51.68% of the total number, more than half of the total number. The number of people aged 31 to 40 is 62, accounting for about 18.96%. There are 96 people aged 41 and above, accounting for 29.36%. From the age point of view, younger employees are more prone to deviant behavior. Education level, 6 people with bachelor degree or below, accounting for 1.83% of the total number. There are 173 undergraduates, accounting for 52.91%. There are 125 graduate students, accounting for 38.23%. There are 23 doctoral students, about 7.03%. From this, it can be seen that the groups engaged in innovative work have certain requirements for academic qualifications, and what they need is only accumulation, and the number of undergraduates and above accounts for the largest proportion.

All procedures of this research were conducted in accordance with the 1964 Declaration of Helsinki and its later amendments or comparable ethical standards. Before conducting this study, the proposals and ethical standards were reviewed and approved by the academic committee of Macau University of Science and Technology. Moreover, we formally introduced to all participants important information about this study and obtained their consent before they participated in the research. Finally, all participant information is anonymous and confidential.

### 3.2 Research tools

Bootleg innovation(Time1): A five-item scale adapted from Criscuolo et al. [12] is used to measure bootleg innovation. Including "I can handle my official work flexibly and tap new potential and valuable opportunities" and "I take the initiative to spend some time to carry out some unofficial projects to enrich future official projects". And using Likert five-point scale method, respectively: 1 = very different, 2 = disagree, 3 = ordinary, 4 = agree, 5 = very agree. Cronbach’s α coefficient of the scale is 0.78.

Emotional intelligence of leaders(Time 2): The 16-item scale of Law et al. [80] was used to measure the emotional intelligence of organizational leaders in this study. Because the questionnaire is filled by employees, it will be expressed in the form of the third person, such as: "He can control his emotions very well, when he is angry, he usually calms down in a short time, and he has a strong ability to control his emotions." Cronbach’s α coefficient of the scale is 0.85.

Cognitive Flexibility(Time 2): Martin & Rubin [69] compiled the Cognitive Flexibility Scale, which is used to evaluate the individual’s cognitive adaptability. The scale has 12 topics. Zhao Bing [81] communicated with Martin, the author of the scale, and revised the cognitive flexibility scale to form a cognitive flexibility scale adapted to the situation in China. The revised scale has 13 topics, such as "I can communicate an idea in many different ways". Among them, there are four reverse topics. Cronbach’s α coefficient of the scale is 0.82.

Individual innovation performance (Time 3): Janssen et al. [82] 9-item scale. Specific topics such as: he often puts forward new methods to create and improve, he often mobilizes support for innovative ideas, and he always takes the initiative to find new working methods, technologies or equipment. Cronbach’s α coefficient of the scale is 0.73.

Control variables: According to previous research, this paper controls the variables that may affect individual innovation performance, namely age, gender and education level. Huang et al. [10] proved that employees’ age, gender and education level will affect their individual innovation performance. Zhao et al. [83] also regarded age, sex and education level as the control variables affecting the relationship between variables.

## 4 Data analysis and research results

### 4.1 Reliability and validity estimates

We also tested the convergence (CR and AVE validity). The results are shown in Table 1. The AVE of all constructs exceeded the baseline 0.05, while the CR exceeded 0.70 [84], indicating good convergence of this study. Validity. In addition, the alpha reliability of the variables is higher than 0.70 (cutoff value).

**Table 1:** Factor loading estimates.

Variables	Factor loading	AVE	CR	Cronbach’s α
*Bootleg innovation*		0.52	0.83	0.78
Item 1	0.78			
Item 2	0.83			
Item 3	0.63			
Item 4	0.62			
Item 5	0.70			
*Cognitive flexibility*		0.59	0.92	0.82
Item 1	0.81			
Item 2	0.83			
Item 3	0.78			
Item 4	0.78			
Item 5	0.77			
Item 6	0.72			
Item 7	0.8			
Item 8	0.77			
Item 9	0.73			
Item 10	0.8			
Item 11	0.72			
Item 12	0.7			
*Leadership emotional intelligence*		0.53	0.90	0.85
Item 1	0.71			
Item 2	0.75			
Item 3	0.71			
Item 4	0.68			
Item 5	0.77			
Item 6	0.7			
Item 7	0.72			
Item 8	0.66			
Item 9	0.8			
Item 10	0.7			
Item 11	0.71			
Item 12	0.78			
Item 13	0.79			
Item 14	0.7			
Item 15	0.66			
Item 16	0.73			
*Individual performance*		0.52	0.82	0.73
Item 1	0.68			
Item 2	0.75			
Item 3	0.72			
Item 4	0.68			
Item 5	0.8			
Item 6	0.66			
Item 7	0.71			
Item 8	0.74			
Item 9	0.69			

### 4.2 Confirmatory factor analysis

To examine discriminate validity, we conducted a confirmatory factor analysis (CFA) on the four self-reported scales, that is, bootleg innovation, Individual innovation performance, cognitive flexibility and emotional intelligence of leaders. Five indices, including *χ^2^*/df, incremental fit index (IFI), Tucker-Lewis index(TLI), comparative fit index(CFI) and root mean square error of approximation(RMSEA) were used to access the model fit. Results showed in Table 2. The four-factor model had good fitting degree (*χ^2^*/df = 1.53, RMSEA = 0.04, TLI = 0.90, CFI = 0.91, IFI = 0.92). At the same time, on the basis of four factors, this study constructed three-factor, two-factor and single-factor models respectively by merging. By comparing with each other, the results showed that the four-factor model had the best indicators and the single-factor model had the worst fitting degree, so it proves that the research constructs had good discrimination validity.

**Table 2:** Confirmatory factor analysis results.

Model	χ^2^	df	χ^2^/df	IFI	TLI	CFI	RMSEA
Four-factor model(A,B,C,D)	1145.56	751	1.53	0.92	0.90	0.91	0.04
Three-factor model(A+B,C,D)	1165.15	754	1.55	0.91	0.89	0.91	0.04
Two-factor model(A+B+C,D)	1707.52	758	2.25	0.80	0.76	0.79	0.06
Single-factor model(A+B+C+D)	3272.09	819	3.99	0.77	0.44	0.46	0.10

Note: A: Bootleg innovation B: Cognitive flexibility C: Leadership emotional intelligence D: Individual innovation performance

### 4.2 Correlation analysis

Table 3 presents the descriptive statistics, including means, standard deviations and correlations for all the variables. According to the results in Table 3, bootleg innovation was positively related to individual innovation performance (*r* = 0.54, *p*<0.001) that provides initial support for *H1*. In addition, in the following analysis, we took gender, age and education level as control variables because they Were correlated with the research variables.

**Table 3:** Correlation analysis.

	Mean	SD	1	2	3	4	5	6
1.Gender	0.77	0.42						
2.Age	34.74	11.20	0.13*					
3.Education level	2.50	0.66	-0.01	-0.03				
4.Bootleg innovation	2.54	0.97	-0.10	-0.13*	0.02			
5.Cognitive flexibility	2.74	0.81	-0.10	-0.06	0.13*	0.76***		
6.Leadership emotional intelligence	2.77	0.70	-0.01	-0.07	0.04	0.17***	0.12***	
7.Individual innovation	2.84	0.60	-0.11*	-0.09	0.13*	0.54***	0.49***	-0.01***

Note: *N* = 327; *p< 0.05, **p < 0.01, ***p < 0.001, ***Significant at *p*<0.01, **Significant at *p*<0.05, *Significant at *p*<0.10

### 4.3 Hypothesis testing

The Process tool for Spss developed by Hayes [85] was used to test all hypotheses. The results in Table 4 showed indicated that bootleg innovation was positively related to individual innovation performance (*b* = 0.33, *p*<0.001,Model1). Thus H1 was supported. In addition, bootleg innovation was

**Table 4:** Regression analysis model.

Variable	Individual innovation performance					Cognitive flexibility	
	Model 1	Model 2	Model 3	Model 4	Model 5	Model 6	Model 7
**Constant**	1.60***	1.58***	1.49***	2.49***	2.62***	0.90***	2.52***
**Control variable**							
Gender	-0.11	-0.11**	-0.10	-0.06	-0.08	-0.05	-0.03
Age	0.01***	0.01*	0.01*	0.01***	0.01*	0.03	0.01
Education level	0.07	0.07	0.07	0.04	0.01	0.06	0.05
**Independent variable**							
Bootleg innovation	0.33***		0.26***	0.21***		0.63**	0.36***
**Mediator**							
Cognitive flexibility		0.35***	0.12*		0.15***		
**Moderator**							
**Leadership emotional intelligence**				0.08**	0.16***		0.06
**Interaction**							
Bootleg innovation × Leadership Emotional Intelligence				0.22***	0.23***		
Cognitive flexibility × leadership emotional intelligence							0.10***
*R^2^*	0.32	0.26	0.34	0.49	0.46	0.58	0.60
ΔR^2^	0.32	0.26	0.34	0.16	0.45	0.58	0.02
*F*	38.64***	28.77***	32.49***	51.05***	44.86***	110.97***	78.44***

Note: *N* = 327; *p< 0.05

**p < 0.01

***p < 0.001

positively related to cognitive flexibility (*b* = 0.63, *p*<0.01, Model 6) and cognitive flexibility was positively related to individual innovation performance(*b* = 0.35, *p*<0.001,Model 2). The results of model 3 further shown that putting bootleg innovation and cognitive flexibility into regression equation simultaneously verifies the mediating effect of cognitive flexibility on bootleg innovation and individual innovation performance. The final result shown that cognitive flexibility has a positive effect on individual innovation performance.

Secondly, in order to verify the mediating effect of cognitive flexibility, Bootstrap test was conducted in this study, and 5000 samples were randomly selected with a confidence interval of 95%. Results As shown in Table 5, the direct effect was 0.26, CI95% = [0.17,0.34], and the indirect effect was 0.08, CI95% = [0.01,0.14], thus it could be seen that cognitive flexibility plays a partial intermediary role between bootleg innovation and individual innovation, and H2 was marginally supported.

**Table 5:** Bootstrap test of mediating effect of cognitive flexibility.

			95%	
	b	Boot SE	The lower limit	The upper limit
Direct effect	0.26	0.04	0.17	0.34
Indirect effect	0.08	0.03	0.01	0.14

**Fig 2:**
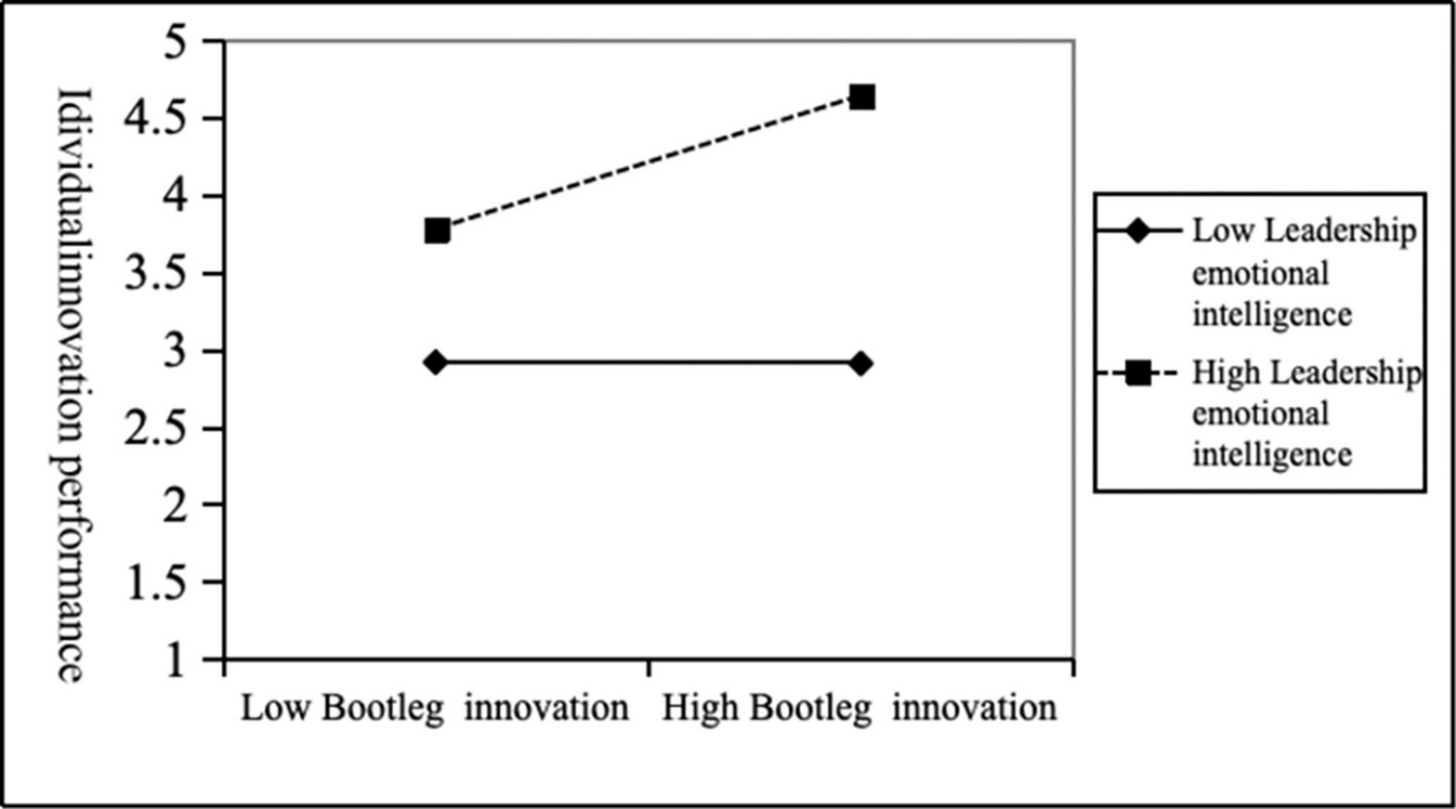
The moderating effect of leadership emotional intelligence on the relationship between bootleg innovation and individual innovation performance.

From the model 4 in Table 4, we could see that the interaction term (bootleg innovation × leadership emotional intelligence) was positively related to individual innovation performance (*b* = 0.22, *p*<0.001), so it was proved that leadership emotional intelligence can promote the positive relationship between bootleg innovation and individual innovation performance, and H3 was supported. Fig 2 shown the moderating effect of leadership emotional intelligence. As can be seen from Fig 2, when the level of leadership emotional intelligence was high, the positive effect of bootleg innovation and individual innovation performance was stronger. When the level of leadership emotional intelligence was low, the positive effect of bootleg innovation and individual innovation performance was weaker. Therefore, as *H3* said, leadership emotional intelligence played a positive moderating role in the positive relationship between bootleg innovation and individual innovation performance.

**Fig 3:**
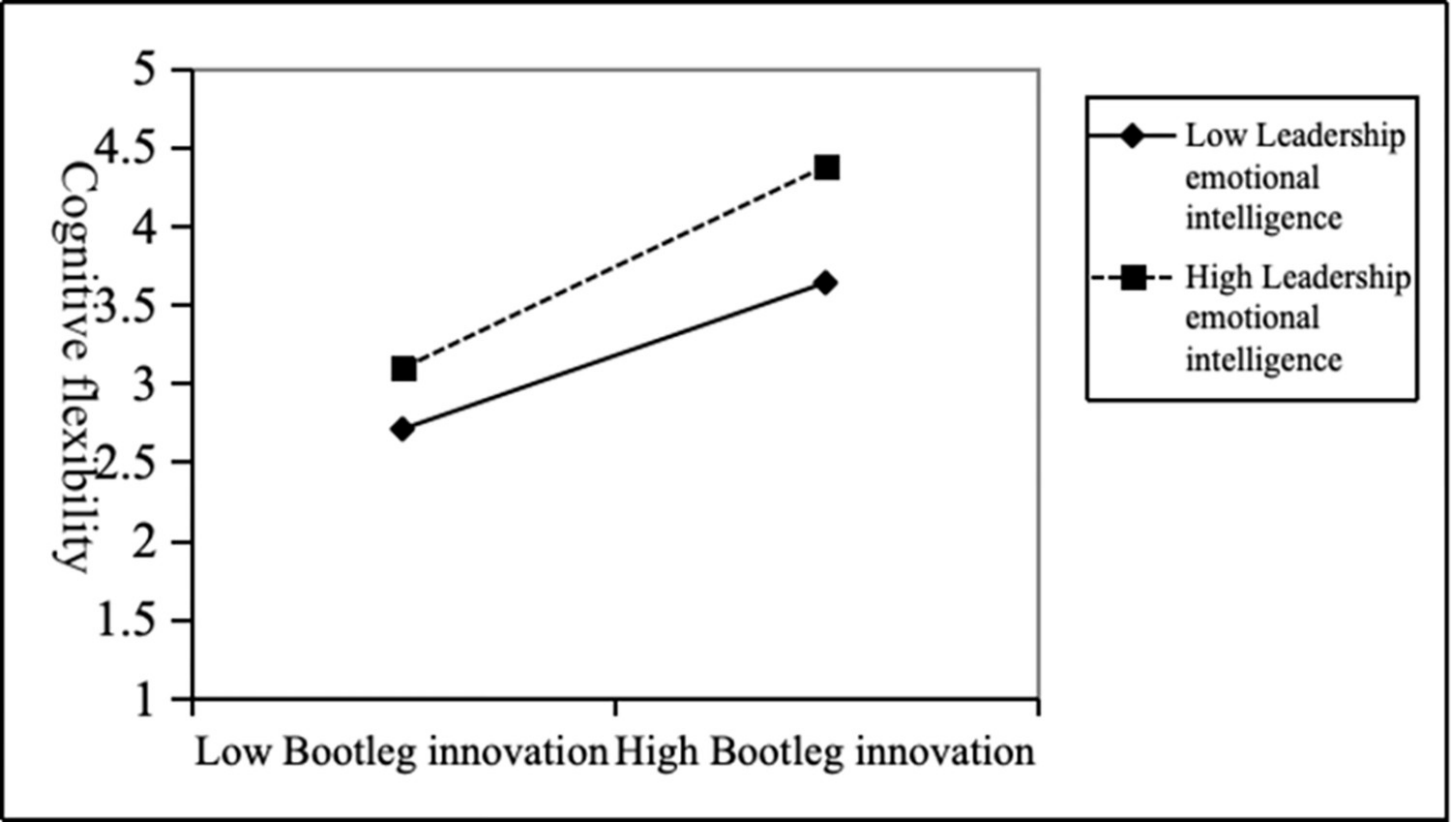
The moderating effect of leadership emotional intelligence on the relationship between bootleg innovation and cognitive flexibility.

Model 5 shown that the interaction between deviant cognitive flexibility and emotional intelligence can promote individual innovation performance (*b* = 23, *p*<0.001). Therefore, leadership emotional intelligence could positively regulate the relationship between bootleg innovation and cognitive flexibility. H4 was supported. At the same time, Fig 3 shown that the higher the emotional intelligence of leaders, the stronger the positive relationship between bootleg innovation and cognitive flexibility. Therefore, H4 was supported.

**Fig 4:**
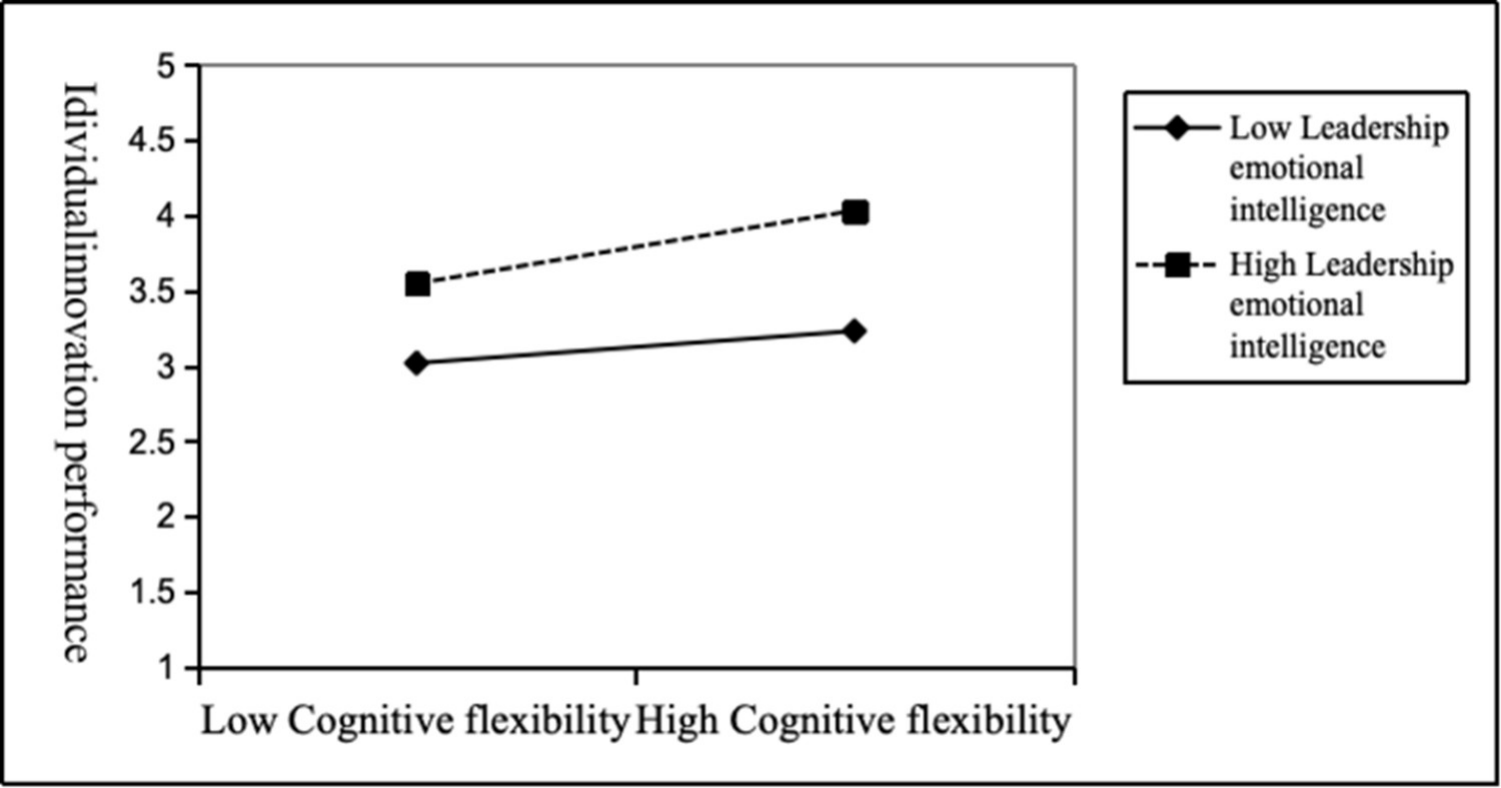
The moderating effect of leadership emotional intelligence on the relationship between cognitive flexibility and individual innovation performance.

Model 7 shown that the interactive item (bootleg innovation × leadership emotional intelligence) was positively related to cognitive flexibility (*b* = 0.10, *p*<0.001), so it was proved that leadership emotional intelligence can promote the positive relationship between bootleg innovation and cognitive flexibility.H5 was supported. Fig 4 shown the moderating effect of leadership emotional intelligence on the relationship between bootleg innovation and cognitive flexibility in a more intuitive way. Fig 4 shown that compared with the low level of leadership emotional intelligence, the high level of leadership emotional intelligence can promote the positive relationship between bootleg innovation and cognitive flexibility. Therefore, H5 was supported. Leadership emotional intelligence positively regulates the relationship between bootleg innovation and cognitive flexibility.

Finally, in order to prove the moderated mediation effect, this study conducted a Bootstrap test, setting 5000 random samples, with a confidence interval of 95%. The results were shown in Table 6. Under the low level of leadership emotional intelligence, the indirect effect was not significant (b = 0.12, CI95% = [0.02,0.22]), and under the high level of leadership emotional intelligence, the indirect effect was significant (b = 0.07, CI95% = [0.01,0.15]). Compared with the indirect effect of lower levels of leadership emotional intelligence, it could be proved that the higher the leadership emotional intelligence, the higher the cognitive flexibility is in the relationship between bootleg innovation and individual innovation performance. The stronger the mediation effect, therefore, H6 was supported.

**Table 6:** Moderated mediating effects.

			95%	
	b	Boot SE	The lower limit	The upper limit
Low Leadership emotional intelligence (-1SD)	-0.06	0.04	-0.14	0.02
High Leadership emotional intelligence (+SD)	0.12	0.05	0.02	0.22

## 5 Research discussion

The current study tested a moderated mediation model in which cognitive flexibility mediated the relationship between bootleg innovation and individual innovation performance, with the relationship between bootleg innovation and individual innovation performance moderated by leader emotional intelligence.

In this study, bootleg innovation is positively related to individual innovation performance. This result is similar to previous research results [86]. That is, employees with bootleg innovation actively and secretly conduct research experiments without formal authorization from their leaders, with the purpose of producing innovative results that are beneficial to the organization [86]. Based on COR theory, individuals may have a tendency to strive to acquire, maintain, cultivate, and protect the resources they value [43]. Therefore, individuals choose to hide innovative ideas, reducing premature exposure and increasing the risk of being rejected by the organization [44]. Individuals can continue to accumulate exploration advantages during innovation activities, providing an important guarantee for later transformation into performance advantages [12]. We further adopted cognitive flexibility as mediator between bootleg innovation and individual innovation performance to discuss the transition states between domains *H2*. Previous research has shown that personal factors are important factors that prompt employees to choose bootleg innovation [27]. Innovation is never easy, and individuals will suffer a steady stream of difficulties. Individuals need to use the only resources, accompanied by strong insight and self-confidence, constantly adjust their perspectives as the environment changes, and actively seek more resources and solutions to overcome problems [24]. Consequently, with the blessing of a flexible cognitive level, it helps individuals to embody more creativity.

As for the moderator, our results are in line with previous studies, claiming leaders’ positive responses to bootleg behavior have a positive effect on the subsequent creative performance of individuals [24, 29]. Social information processing theory describes that an individual’s behavior and activities are influenced by the surrounding environment [87, 88]. Leaders with high emotional intelligence are better at perceiving, evaluating and expressing emotions, and handling the relationship between themselves and employees well [68]. *H3* presents that leadership emotional intelligence may decrease the impact of bootleg innovation on individual innovation performance. Stress and failure in the innovation process will cause individuals to feel frustrated, and relying on individual self-regulation alone is not enough to revive them. Support and encouragement from leaders can give employees more motivation. For leaders with high emotional intelligence, on the one hand, they are good at controlling their own emotions and not being slaves of emotions; on the other hand, they can accurately grasp the emotions of others and provide appropriate support [57], so as to motivate employees to continue to work hard and achieve goals. *H4* describes that leadership emotional intelligence may strengthen the positive relationship between bootleg innovation and cognitive flexibility. It demonstrates that leadership emotional intelligence as a moderator in between bootleg innovation and cognitive flexibility may re-emphasize the positive effects of leadership factors on bootleg innovation behavior and individual employee emotions [28]. *H5* proposes that leadership emotional intelligence may strengthen the positive relationship between cognitive flexibility and individual innovation performance. It can be seen that leaders are good at capturing employees’ emotional changes and giving positive feedback, which can help employees establish an optimistic and positive attitude to solve problems and stimulate creativity [45]. *H6* proposes that leadership emotional intelligence may further influence the indirect relationship between bootleg innovation and individual innovation performance through cognitive flexibility. In accordance with previous research, when leaders and individuals have a high degree of cognitive fit, individuals will have better work behaviors and outcomes [74]. Although bootleg innovation is subject to enormous pressure, leadership emotional intelligence can well relieve employees’ negative emotions when they encounter setbacks. Individuals are more likely to change their minds mentally and ideologically, solve current problems and achieve good individual innovation performance [77].

## 6 Theoretical implications and practical implications

### 6.1 Theoretical implications

This study explores the positive impact of bootleg innovation on individual innovation performance from the COR theory and social information processing theory, and challenges the stereotype that "bootleg innovation" is bootleg in the existing thinking. The academic community has not reached a consensus on whether bootleg innovation can bring benefits to employees. Scholars with a positive view believe that individuals who bootleg from innovation based on self-efficacy and saving the time and energy of reporting to leaders can increase work engagement and successfully transform exploration advantages into performance advantages [7, 12]. Scholars with negative views believe that bootleg innovation behavior is a challenge to organizational rules and regulations, and once discovered, it will be punished, which is not conducive to individual innovation performance [88]. However, most of these inferences remain at the theoretical level and lack sufficient empirical data support. The impact of bootleg innovation on individual innovation performance is influenced by many factors [17]. Simply defining bootleg innovation as "good soldiers" or "bad apples" is biased. For organization, how to control bootleg innovation, develop advantages, and turn them into "good soldiers" is the most important task. By proving that individual factors (cognitive flexibility) and leadership factors (leadership emotional intelligence) can promote bootleg innovation and individual innovation performance, this study enriches the research on the effects of deviant innovation behavior on the one hand, and on the other hand, through quantitative evidence effectively elucidates the boundary conditions for bootleg innovation and individual innovation performance.

This study incorporated leadership emotional intelligence as a moderating variable into the research model. It is proved that in the organization, the surrounding environment and the emotional value of the leader will affect the behavior and state of employees, which echoes the previous research [75]. It is worth noting that although leadership factors are also used as moderating variables in recent studies to explore the path of deviant behavior. For example, Li et al. [45] found that leadership feedback behavior will be used as a criterion for employee creativity, and positive feedback can improve creativity. Negative feedback reduces creativity. However, this study is significantly different from that study. This study explores the impact of emotional value provided by leaders on individual bootleg innovation. Emotional stability of leaders, together with leaders’ mastery and understanding of employees’ emotional changes, has the effect of revitalizing employees [55]. It can be seen that this study is significantly different from the existing research on the influence of leadership factors on deviant innovation. Furthermore, this study takes cognitive flexibility as an mediator. Many previous studies used cognitive flexibility as an important measurement tool to study students’ learning, and it was seldom used in organizational research [89]. This study enriches the research on cognitive flexibility in the field of organization.

Finally, this study holds that bootleg innovation is a unique research method, and many scholars have held different views for a long time. In view of the frontier, concealment and illegality of bootleg innovation, many scholars believe that bootleg innovation should be avoided in organizations that have no rules and regulations [36], but it has been repeatedly banned in enterprises that aim at innovation. Because it is the most important thing to give full play to the advantages of bootleg innovation. At the same time, it provides a new idea for further discussing how to promote the positive effect of bootleg innovation on individual innovation performance and avoid the occurrence of negative effects.

### 6.2 Practical implications

The conclusions of this study have important implications for organizational management practice. Organizations need to value innovation. In other words, organizations need to take a comprehensive and dialectical view of bootleg innovation behaviors in their organizations. Many existing studies have found that bootleg innovation has a positive impact on individual performance or team performance [16, 19]. These studies show that bootleg innovation has positive and important meanings to individual and organizational development to a certain extent. Making good use of employees’ bootleg innovation can bring benefits to the organization and improve the level of organizational innovation. On the one hand, bootleg innovation in an organization can increase competitive advantage and promote product innovation to a certain extent. On the other hand, bootleg innovation behavior may promote the emergence of organizational citizenship behavior to a certain extent [36]. Therefore, rational use of bootleg innovation by the organization will bring huge benefits to the organization. Specific suggestions are as follows:

To avoid hasty rejection of employees’ new innovations, there should be a more scientific and reasonable judgment mechanism. Reduce the probability of missing valuable innovations. Deviant behavior will indeed increase employees’ energy consumption. Organizationally, a fair, just and transparent evaluation link can reduce employees’ deviant innovative behavior. Carrying out innovation work with the support of organizations is often more likely to produce positive innovation effects than bootleg innovation [90].

Create a harmonious and positive innovation atmosphere for employees. In the process of innovation, employees will encounter all kinds of difficulties. Whether it is the approved idea or the bootleg innovation process, the understanding and concern of the leaders, timely detecting the changes in employees’ mood, comforting and supporting them, have positive effects on the innovation process of employees[88].

Good psychological quality and flexible thinking mode are very important for employee innovation research [91]. In the face of difficulties, actively looking for other methods and adjusting individual pressure have a positive effect on improving innovation performance. Therefore, employees should cultivate flexible cognitive ability, actively communicate with colleagues and leaders, and build a good interactive environment, thus contributing to the implementation of creativity.

## 7 Research limitations and future prospects

This study has some limitations that shed light on future research directions. First, though our findings provide an integrated view for bootleg innovation research. The individual innovation performance questionnaire was filled in by the employee’s immediate leader, and the rest of the variables were measured by means of self-reports. Considering the possibilities of common methods variance, the survey was conducted via three-stage process. While distributing the questionnaire, it was emphasized that this survey was conducted in an anonymous manner and was only used for research. However, bootleg innovation has a certain sensitivity in the workplace, and self-reporting by employees may result in concealment.

Secondly, the national conditions of China and Western countries are very different. There is an old saying that "you can’t make a circle without rules", and China and Western countries advocate different etiquette. The scale adopted for bootleg innovation is adapted from the scale of Criscuolo et al. [12]. Although it is a well-developed scale, there may be uncertainties in measuring the behavior of Chinese employees. Therefore, in terms of bootleg innovation, we can try to develop a scale based on China’s national conditions and culture in the future.

Thirdly, the study focuses on the impact of bootleg innovation on individual innovation performance, and constructs a model with cognitive flexibility as the mediator and leadership emotional intelligence as the moderator, then empirical research proves the value of this path. For a long time, more and more organizations hope to use the team form to integrate the knowledge and skills of team members, so as to effectively improve productivity and economic benefits [92]. Therefore, whether this factor also promotes the relationship between bootleg innovation and team performance remains to be explored.

Finally, this study only conducts empirical research with cognitive flexibility as a mediator and leader emotional intelligence as a moderator. Situational factors (employee political skills) and organizational climate (workplace friendship) and other factors can help employees obtain other colleagues’ perceptions of bootleg innovation. Tolerance and understanding, the path of action between them is worthy of our further exploration [36].

## 8 Conclusion

In this study, we address the controversy over the impact of bootleg innovation on individual innovation performance. The role of bootleg innovation is restricted by many factors. Bootleg innovation further promotes sustainable performance and establishes advantages through the mechanism of cognitive flexibility on individual innovation performance. Rather than blindly suppressing bootleg innovation. Organizations must consider different ways to develop the advantage of bootleg innovation. Therefore, this study will include cognitive flexibility as a mediator between bootleg innovation and individual innovation performance, and use leadership emotional intelligence as a moderator to promote the development of bootleg innovation in a good direction through the joint action of individual factors and leadership factors (individual innovation performance). By introducing coping mechanisms, practices, and interventions, they may help promote the positive side of bootleg innovation.

## References

[1] Antoncic B. Intrapreneurship: a comparative structural equation modeling study. Industrial Management & Data Systems. 2007; 107: 309–325. 10.1108/02635570710734244

[2] Ullah S, Ahmad T, Lyu B, Sami A, Kukreti M, Yvaz A. Integrating external stakeholders for improvement in green innovation performance: role of green knowledge integration capability and regulatory pressure. International Journal of Innovation Science. 2023;(ahead-of-print). 10.1108/IJIS-12-2022-0237

[3] Games D, Hidayat T, Fhardilha J, Fernando Y, Sari DK. The impact of trust, knowledge sharing, and affective commitment on SME innovation performance. Journal of Governance and Integrity. 2022; 5: 267–274. 10.15282/jgi.5.2.2022.7184

[4] Khan M, Raya RP, Viswanathan R. Enhancing employee innovativeness and job performance through a culture of workplace innovation. International Journal of Productivity and Performance Management. 2021; 71: 3179–3204. 10.1108/IJPPM-09-2020-0466

[5] Jiang Y, Jiang Y, Nakamura W. Human capital and organizational performance based on organizational innovation: Empirical study on China. Revista de Cercetare Si Interventie Sociala. 2019; 64:156–166. 10.33788/rcis.64.13

[6] Luo M, Zhao L, Lyu B. Exploring the fuzzy integrated assessment of college students’ education for innovative entrepreneurship under the background of internet+. Security and Communication Networks. 2022; 1–12. 10.1155/2022/4339772

[7] Mainemelis C. Stealing fire: Creative deviance in the evolution of new ideas. Academy of management review. 2010; 35: 558–578. 10.5465/amr.35.4.zok558

[8] O’Connor GC, DeMartino R. Organizing for radical innovation: An exploratory study of the structural aspects of RI management systems in large established firms. Journal of product innovation management. 2006; 23: 475–497. 10.1111/j.1540-5885.2006.00219.x

[9] Augsdorfer P. Bootlegging and path dependency. research policy. 2005; 34: 1–11. 10.1016/j.respol.2004.09.010

[10] Huang W, Xiang GP, Du YZ, Liu Y. Bootleg and individual innovation performance: the joint effect of status and creativity. Nankai Business Review. 2007; 1: 143–154. 10.3969/j.issn.1008-3448.2017.01.013

[11] Wang XL, Wang MY, Liu JN. Study on the influence mechanism of leaders’ abusive supervision on employees’ bootlegging innovation behavior. International Journal of Conflict Management. 2023; 32:887–906. 10.1108/IJCMA-02-2023-0026

[12] Criscuolo P, Salter A, Ter Wal AL. Going underground: Bootlegging and individual innovative performance. Organization Science. 2014; 25: 1287–1305. 10.1287/orsc.2013.0856

[13] Dahling JJ, Gutworth MB. Loyal rebels? A test of the normative conflict model of constructive deviance. Journal of Organizational Behavior. 2017; 38: 1167–1182. 10.1002/job.2194

[14] Huang W, Xiang G, Du Y, Liu Y. Bootlegging and Individual Innovation Performance: The Joint Effect of Status and Creativity. In Corporate Underground: Bootleg Innovation and Constructive Deviance. 2022; 241–246. 10.1142/9781800612266_0011

[15] Zheng X, Mai S, Zhou C, Ma L, Sun X. As above, so below? The influence of leader humor on bootleg innovation: The mechanism of psychological empowerment and affective trust in leaders. Frontiers in Psychology. 2022;13–956782. doi: 10.3389/fpsyg.2022.956782 36186310 PMC9524421

[16] Cui Z, Wang HY, Zhao D. Impact of Constructive Deviance on Individual Creative Performance Moderating Effect of Internal Social Capital. R&D Management. 2020; 32: 63–71. 10.13581/j.cnki.rdm.20181026

[17] Globocnik D, Peña Häufler B, Salomo S. Organizational antecedents to bootlegging and consequences for the newness of the innovation portfolio. Journal of Product Innovation Management. 2020; 39: 717–745. 10.1111/jpim.12626

[18] Ye P, Liu L, Tan J. Creative leadership, innovation climate and innovation behaviour: the moderating role of knowledge sharing in management. European Journal of Innovation Management. 2022; 25: 1092–1114. 10.1108/EJIM-05-2020-0199

[19] Yang G, Song JM, Ji HP. Employee Creativity and Creative Deviance: Based on the research of Psychological Entitlement and Moral Disengagement. Science & Technology Progress and Policy. 2019; 35: 115–122. 10.6049/kjjbydc.L201808581

[20] Han DH, Park HW, Kee BS, Na C, Na DHE, Zaichkowsky L. Performance enhancement with low stress and anxiety modulated by cognitive flexibility. Psychiatry investigation. 2011; 8: 221–226. doi: 10.4306/pi.2011.8.3.221 21994509 PMC3182387

[21] Cragg L, Chevalier N. The processes underlying flexibility in childhood. Quarterly Journal of Experimental Psychology. 2012; 65:209–232. doi: 10.1080/17470210903204618 19921594

[22] Madjar N, Greenberg E, Chen Z. Factors for radical creativity, incremental creativity, and routine, noncreative performance. Journal of applied psychology. 2011;6: 730–743. doi: 10.1037/a0022416 21319879

[23] Miron-Spektor E, Ingram A, Keller J, Smith WK, Lewis MW. Microfoundations of organizational paradox: The problem is how we think about the problem. Academy of Management Journal. 2018; 61: 26–45. 10.5465/amj.2016.0594

[24] Wu DY, Gao LL, Duan JY. The Mechanism of Job Engagement on Voice Behavior: The Moderating Effects of Cognitive Flexibility and Power Motive. Chinese Journal of Applied Psychology. 2014; 20: 67–75. 10.3724/sp.j.1041.2021.00199

[25] Salancik GR, Pfeffer J. A social information processing approach to job attitudes and task design. Administrative science quarterly. 1978;23: 224–253. 10.2307/2392563 10307892

[26] Wang W, Liu HQ. Research on Antecedents, Processes and Results of Deviant Innovation. Science and Technology Management Research. 2020; 15:20–25. 10.3969/j.issn.1000-7695.2020.15.003

[27] Augsdorfer P. A diagnostic personality test to identify likely corporate bootleg researchers. International Journal of Innovation Management. 2012; 16: 12500031–125000318. 10.1142/S1363919611003532

[28] Dasborough MT, Ashkanasy NM. Emotion and attribution of intentionality in leader–member relationships. The Leadership Quarterly. 2002;13: 615–634. 10.1016/S1048-9843(02)00147-9

[29] Su W, Lyu B, Chen H, Zhang Y.How does servant leadership influence employees’ service innovative behavior? The roles of intrinsic motivation and identification with the leader. Baltic Journal of Management. 2020; 15: 571–586. 10.1108/BJM-09-2019-0335

[30] Sun G, Lyu B. Relationship between emotional intelligence and self-efficacy among college students: the mediating role of coping styles. Discover Psychology. 2022; 2: 42. 10.1007/s44202-022-00055-1

[31] George JM. Emotions and leadership: The role of emotional intelligence. Human relations. 2000; 53:1027–1055. 10.1177/0018726700538001

[32] Rajah R, Song ZL, Arvey RD. Emotionality and leadership: Taking stock of the past decade of research. The Leadership Quarterly. 2011; 22: 1107–1119. 10.1016/j.leaqua.2011.09.006

[33] Nguyen NT, Hooi LW, Avvari MV. Leadership styles and organisational innovation in Vietnam: does employee creativity matter?. International Journal of Productivity and Performance Management. 2023; 72: 331–360. 10.1108/IJPPM-10-2020-0563

[34] Gajdzik B, Wolniak R. Smart production workers in terms of creativity and innovation: The implication for open innovation. Journal of Open Innovation: Technology, Market, and Complexity 2022; 8: 68. 10.3390/joitmc8020068

[35] Warren DE. Constructive and destructive deviance tn organizations. Academy of management Review.2003; 28: 622–632. 10.5465/amr.2003.10899440

[36] Wang YZ, Zhang T. The Double-Edged Sword Effect of Constructive Deviance on Individual Innovation Performance. Contemporary economic management.2020; 42:73–78. 10.3389/fpsyg.2022.892395

[37] Burgelman RA, Sayles LR. Inside Corporate Innovation: Strategy, Structure, and Managerial Skills (Free Press, New York). 1986. 10.3917/mana.182.0179

[38] O’Connor GC, McDermott CM.The human side of radical innovation. Journal of engineering and technology management. 2004; 21:11–30. 10.1016/j.jengtecman.2003.12.002

[39] March JG. Exploration and exploitation in organizational learning. Organization science. 1991; 2: 71–87. 10.1287/orsc.2.1.71

[40] Garud R, Gehman J, Kumaraswamy A. Complexity arrangements for sustained innovation: Lessons from 3M Corporation. Organization Studies. 2011; 32: 737–767. 10.1177/0170840611410810

[41] Koch R, Leitner KH. The dynamics and functions of self‐organization in the fuzzy front end: Empirical evidence from the Austrian semiconductor industry. Creativity and Innovation Management.2008; 17: 216–226. 10.1111/j.1467-8691.2008.00488.x

[42] Spreitzer GM, Sonenshein S. Toward the construct definition of positive deviance. American behavioral scientist. 2004; 47: 828–847. 10.1177/0002764203260212

[43] Hobfoll SE. Conservation of resources: A new attempt at conceptualizing stress. American psychologist. 1989; 44: 513–524. 10.1037/0003-066X.44.3.513 2648906

[44] March JG. Rationality, foolishness, and adaptive intelligence. Strategic management journal. 2006; 27: 201–214. 10.1002/smj.515

[45] Li XM, Xu ZT, Huo WW. The influence of Creative Deviant Behavior to Creativity: Moderating Effect of Leadership Feedback and the Mediating Effect of Creative Self-Efficacy. Science & Technology Progress and Policy. 2019; 36: 138–145. 10.6049/kjjbydc.2018110430

[46] Martin MM, Anderson CM. The relationship between cognitive flexibility and affinity-seeking strategies. Advances in Psychological Research.2001; 4: 69–76. 10.14689/ejer.2018.77.6

[47] Martin MM, Anderson CM. The cognitive flexibility scale: Three validity studies. Communication Reports. 1998; 11: 1–9. 10.1080/08934219809367680

[48] Zhou M, Yu K, Wang FR. Cognitive Flexibility and Individual Adaptability: A Cross-Lag Bidirectional Mediation Model. Chinese Journal of Clinical Psychology. 2021; 29: 182–190. 10.16128/j.cnki.1005-3611.2021.01.037

[49] Luo JL, Hu WA, Zhong J. Perception of Organizational Change Climate and Commitment to Organizational Change: A Moderating Model. Management Review. 2017; 29:122–134. 10.14120/j.cnki.cn11-5057/f.2017.06.007

[50] Zhang MN, Wang JT, Que MK. Does Innovation and Entrepreneurship Competition Promote the Entrepreneurial Intention of Higher Vocational College Students?——An Empirical Research Based on the Perspective of Cognitive Flexibility and Entrepreneurial Alertness. Vocational and Technical Education. 2022; 5: 75–80. http://doi.org/ 10.3969/j.issn.1008-3219.2022.05.017

[51] Tepper B, Dimotakis N, Lambert LS, Koopman J, Matta FK, Man Park H, et al. Examining follower responses to transformational leadership from a dynamic, person–environment fit perspective. Academy of Management Journal.2018; 61:1343–1368. 10.5465/amj.2014.0163

[52] Liu ZQ, Yan RX, Tang SS. Leader innovation expectations and employee’s breakthrough innovation engagement: a study based on paradox theory. Management World. 2021; 37:226–241. 10.19744/j.cnki.11-1235/f.2021.0166

[53] Kiss AN, Libaers D, Barr PS, Wang T, Zachary MA. CEO cognitive flexibility, information search, and organizational ambidexterity. Strategic Management Journal. 2020; 41: 2200–2233. 10.1002/smj.3192

[54] Woodman RW, Sawyer JE, Griffin RW. Toward a theory of organizational creativity. Academy of management review.1993; 18: 293–321. 10.5465/amr.1993.3997517

[55] Yu Q, Yuan DH. The Impact of the Emotional Intelligence of Employees and Their Manager on the Job Performance of Employees. Acta Psychologica Sinica. 2008; 1: 74–83. 10.7666/d.y1223749

[56] Sy T, Tram S, O’hara LA. Relation of employee and manager emotional intelligence to job satisfaction and performance. Journal of vocational behavior. 2006; 68: 461–473. 10.1016/j.jvb.2005.10.003

[57] Wong CS, Law KS. The effects of leader and follower emotional intelligence on performance and attitude: An exploratory study. The Leadership Quarterly.2002; 13: 243–274. 10.1016/S1048-9843(02)00099-1

[58] Luo JL, Li SW, Liang F. The route and Condition of Effect of Leaders’ Emotional Intelligence Congruence on Employee Voice. Management Review. 2022; 34: 198–208. 10.14120/j.cnki.cn11-5057/f.20210210.007

[59] McClelland DC. Identifying competencies with behavioral-event interviews. Psychological science. 1998; 9: 331–339. 10.1111/1467-9280.00065

[60] Qi DW, Wu XD. Good Teachers and Helpful Friends Persuade Employees to Stay?. Human Resource and Development of China. 2015; 56–61. 10.16471/j.cnki.11-2822/c.2015.09.008

[61] Shi K, Song X, Zhou R, Zhou W. The effect of team cultural tightness and transformational leadership on employee creative behavior: A cross‐level moderated mediation model. PsyCh Journal. 2023; 12: 657–669. doi: 10.1002/pchj.678 37681242

[62] Song Y, Shi DD. Deviant Innovation, Employee Autonomy and Organizational Innovation Ability. Journal of Harbin University of Commerce (Social Science Edition). 2022; 84–93. http://doi.org10.3969/j.issn.1671-7112.2020.04.007

[63] Martin MM, Rubin RB. A new mresure of cognitive flexibility. Psychological Reports. 2011; 623–626. 10.2466/pr0.1995.76.2.623

[64] Jiang YJ, Xu YH. What Exactly is Bootleg Innovation:A Literature Review And Future Research Agenda. Science & Technology Progress and Policy. 2023; 40:150–160. 10.6049/kjjbydc.2022050208.

[65] Antonakis J, Ashkanasy NM, Dasborough MT. Does leadership need emotional intelligence?. The leadership quarterly. 2009; 20: 247–261. 10.1016/j.leaqua.2009.01.006

[66] Rong Y, Sui Y, Yang BY. The Effect of Leader Emotional Intelligence on Group Performance and Employee Attitude: Mediating Effect of Justice Climate and Moderating Effect of Group Power Distance.ActaPsychologicaSinica. 2015; 47: 1152–1161. http://doi.org10.3724/SP.J.1041.2015.01152

[67] Sok KM, Sok P, Tsarenko Y, Widjaja JT. How and when frontline employees’ resilience drives service-sales ambidexterity: the role of cognitive flexibility and leadership humility. European Journal of Marketing.55. 2021; 2965–2987. 10.1108/EJM-05-2020-0320

[68] Mayer JD, Salovey P. What is emotional intelligence? In Salovey P, & Sluyter D (Eds.), Emotional development and emotional intelligence: Educational implications (pp. 3–34). New York: Basic Books. 1997; 3–34. 10.12691/education-1-6-8

[69] Martin MM, Rubin RB. A new measure of cognitive flexibility. Psychological reports. 1995; 76: 623–626. 10.2466/pr0.1995.76.2.623

[70] Tjosvold D, Johnson DW, Johnson RT, Sun H. Competitive motives and strategies: Understanding constructive competition. Group Dynamics: Theory, Research, and Practice. 2006; 10: 87–99. 10.1037/1089-2699.10.2.87

[71] Shin SJ, Kim TY, Lee JY, Bian L. Cognitive team diversity and individual team member creativity: A cross-level interaction. Academy of management journal. 2012; 55:197–212. 10.5465/amj.2010.0270

[72] Suliman AM, Al‐Shaikh FN. Emotional intelligence at work: Links to conflict and innovation. Employee relations. 2007; 29: 208–220. 10.1108/01425450710720020

[73] Kafetsios K, Nezlek JB, Vassiou A. A multilevel analysis of relationships between leaders’ and subordinates’ emotional intelligence and emotional outcomes. Journal of Applied Social Psychology. 2011; 41: 1121–1144. 10.1111/j.1559-1816.2011.00750.x

[74] Kristof‐Brown AL, Zimmerman RD, Johnson EC. Consequences OF INDIVIDUALS’FIT at work: A meta‐analysis OF person–job, person–organization, person–group, and person–supervisor fit. Personnel psychology. 2005; 58: 281–342. 10.1111/j.1744-6570.2005.00672.x

[75] Lv HJ, Han CX, Wang DJ. The relationship between emotional intelligence and leadership effectiveness: A meta-analysis. Advances in Psychological Science. 2018; 26: 204–220. 10.1080/0305764X.2021.1927987

[76] Tang CY, Pan Y.The Impact of Emotional Intelligence and Organizational Identification on Organizational Citizenship Behavior: A Multilevel Analysis. Nankai Business Review. 2010; 13: 115–124. 10.1061/41127(382)12

[77] Xu SY, Zhu JQ. Ethical leadership and pro-social rule breaking: A dual process model. Acta Psychologica Sinica. 2017; 29: 106–115. 10.3724/SP.J.1041.2017.00106

[78] Sun LY, Aryee S, Law KS. High-performance human resource practices, citizenship behavior, and organizational performance: A relational perspective. Academy of management Journal. 2007; 50: 558–577. 10.5465/amj.2007.25525821

[79] Podsakoff NP. Common method biases in behavioral research: a critical review of the literature and recommended remedies. Journal of applied psychology. 2003; 88: 879–903. doi: 10.1037/0021-9010.88.5.879 14516251

[80] Law KS, Wong CS, Song LJ. The construct and criterion validity of emotional intelligence and its potential utility for management studies. Journal of Applied Psychology. 2004; 89: 483–96. doi: 10.1037/0021-9010.89.3.483 15161407

[81] Qi B, Zhao B, Wang K, Liu HH. Revision and Preliminary Application of Cognitive Flexibility Scale for College Students.Studies of Psychology and Behavior. 2012; 11: 120–123. https://psybeh.tjnu.edu.cn/EN/Y2013/V11/I1/120

[82] Janssen O, Van Yperen NW. Employees’ goal orientations, the quality of leader-member exchange, and the outcomes of job performance and job satisfaction. Academy of management journal. 2004; 47: 368–384. 10.5465/20159587

[83] Zhao FQ, Zhou Q, Chen Y. Impact of international and external integrated development on individual innovation performance——The role of knowledge manipulation and proof goal orientation. Science Research Management. 2022; 43:124–133. 10.19571/j.cnki.1000-2995.2022.11.013

[84] Hair JF, Black WC, Babin BJ, Anderson RE. Multivariate Data Analysis: A Global Perspective, Pearson Prentice Hall, New Jersey. 2010. 10.1080/24749508.2018.1504272

[85] Hayes AF. PROCESS: A versatile computational tool for observed variable mediation, moderation, and conditional process modeling. Guilford Press. 2012; 1–39. http://www.afhayes.com

[86] Nanyangwe CN, Wang H, Cui Z. The end justifies the means: the role of organizational identification on bootleg innovation behavior. Journal of Management & Organization. 2023; 1–16. 10.1017/jmo.2022.92

[87] Walther JB. Social information processing theory (CMC). The international encyclopedia of interpersonal communication. 2015; 1–13. 10.1002/9781118540190.wbeic192

[88] Globocnik D, Salomo S. Do formal management practices impact the emergence of bootlegging behavior?. Journal of Product Innovation Management. 2015; 32: 505–521. 10.1111/jpim.12215

[89] Deák GO, Wiseheart M. Cognitive flexibility in young children: General or task-specific capacity?. Journal of experimental child psychology. 2015; 138: 31–53. doi: 10.1016/j.jecp.2015.04.003 26026421

[90] Jiang Y, Chen CC. Integrating knowledge activities for team innovation: Effects of transformational leadership. Journal of Management. 2018; 44: 1819–1847. 10.1177/0149206316628641

[91] Barańczuk U. The Five Factor Model of personality and social support: A meta-analysis. Journal of Research in Personality. 2019; 81: 38–46. 10.1016/j.jrp.2019.05.002

[92] Guzzo RA, Dickson MW. Teams in organizations: Recent research on performance and effectiveness. Annual review of psychology. 1996; 47: 307–338. doi: 10.1146/annurev.psych.47.1.307 15012484

